# Evaluation of neutrophil/lymphocyte ratio and platelet/lymphocyte ratio in recurrent epistaxis in childhood: case controlled study

**DOI:** 10.11604/pamj.2019.32.154.18372

**Published:** 2019-04-02

**Authors:** Ceyhun Aksakal, Mehmet Şahin

**Affiliations:** 1 Tokat State Hospital, Department of Otolaryngology, Tokat, Turkey; 2 Tokat State Hostital, Department of Biochemistery, Tokat, Turkey

**Keywords:** Epistaxis, children, neutrophil-to-lymphocyte ratio, platelet-to-lymphocyte ratio

## Abstract

**Introduction:**

the aim of this study was to investigate the possible relationship of neutrophil-lymphocyte ratio (NLR) and platelet-lymphocyte ratio (PLR) and routine hematological parameters with recurrent epistaxis in children.

**Methods:**

İn this retrospective case-controlled study, 294 patients aged between 2 and 18 years who applied to the Tokat State Hopital Ear Nose Throat Clinic due to recurrent epistaxis between January 1^s^t 2013 and December 31^st^ December 2017 and 329 sex-and age-matched controls were investigated.

**Results:**

NLR was 1.45±0.75 in the study group and the 1.35±0.7 in the control group. There was no significant difference between the groups (p>0.05). PLR values were found significantly (p<0.05) higher in the study group than in the control group (103,21±29.57 vs. 97,3±30.38). Red Blood Cell Distribution Width (RDW) values were found significantly (p<0.05) lower in the study group than in the control group (39,56±2,87 and 38,92±2,46).

**Conclusion:**

the increase of PLR, an inflammatory marker, in epistaxis supports the effect of inflammatory factors in the etiology of epistaxis. However, more study in future is needed to support this.

## Introduction

Epistaxis is a condition often seen in childhood and it’s rarely seen before 2 years of age. It was reported that the epistaxis affects 30% of children under 5 years and 50% of children older than 5 years of age [1]. Epistaxis in childhood is a condition that is self-limiting and rarely requires a nasal tamponade [2]. Epistaxis may originate from anterior and posterior according to source of bleeding. Usually, the nosebleeds originated from anterior septum called Little's area (also known as Kiesselbach's plexus) which are seen in children [3]. In the epistaxes of childhood, it is argued that the etiology is known in very few cases. Blood dyscrasia, blood vessel problems with telangiectatic vessels in the Little's area, and diseases causing bleeding disorder (von Willebrand's disease, Idiopathic Thrombocytopenic Purpura (ITP) are among the known reasons of epistaxis. In majority of cases of epistaxis, bleeding is originated from the veins in the anterior septum and sometimes from arteries. This last condition is known as “Idiopathic epistaxis” [1]. Although the tests such as prothrombin time (PT), activated partial thromboplastin time (aPTT) and international normalized ratio (INR) are the most important diagnostic parameters in the investigation of possible coagulation disorders, the levels of these parameters are normal in most of the levels of epistaxis [4].

Neutrophil lymphocyte ratio (NLR) and platelet to lymphocyte ratio (PLR) are recently used to evaluate especially systemic inflammation [5]. NLR is used as an easily accessible and inexpensive prognostic test tool in major cardiac conditions, ischemic stroke, sepsis and infectious diseases and its efficiency was proven [6-8]. It was shown that the nasal colonization of *Staphylococcus aureus* was higher in children with recurrent epistaxis than the control group [9]. It was claimed that the nasal colonization of *Staphylococcus aureus* stimulates the inflammation, and the risk of crusting, neo-vascularization and increased recurrent epistaxis occured in the nasal septum due to this condition [10]. This situation brought the possible effect of inflammation on epistaxis to forefront. In previous studies, different blood parameters were investigated in pediatric recurrent epistaxis [11, 12]. In our study, we aimed to investigate especially inflammatory markers NLR, PLR, and some other blood parameters in pediatric recurrent epistaxis.

## Methods

The study was approved by the Gaziosmanpaşa University Medical School Clinical Research Ethics Committee. (Approval Number: 18-KAEK-237). Two hundred and ninety four patients aged between two and 18 years who applied to the Tokat State Hospital. Outpatient Clinic due to recurrent epistaxis between January 2013 and December 2017 were included in this retrospective, case-controlled study. The control group consisted of 329 age and sex matched pediatric cases who underwent the inguinal hernia repair and circumcision at the same period and who did not have any disease. Recurrent epistaxis was defined as the nosebleeding which occurred at least twice a week. The inclusion criteria for the study group and the control group are: (a) patients with recurrent epistaxis, (b) digital trauma (c) patients without any coagulation disorder and patients not receiving anticoagulant therapy, (d) patients without any systemic disease which may be the cause of bleeding, (e) patients with acute infection, (f) patients with chronic systemic disease. Patients with abnormal blood results such as anemia and leukocytosis were excluded from study.

Blood samples of the study and control groups were obtained from the antecubital vein. Automated blood cell counter (Mindray BC-6800, Guangdong, China) was used in the measurement of complete blood count (CBC). Mean values found in CBC were used for statistical evaluation. With CBC test results, NLR value was obtained by dividing absolute neutrophil count by absolute lymphocyte count, and PLR value was obtained by dividing the absolute platelet count by absolute lymphocyte count. These values were used in statistical evaluation.

**Statistical analysis:** statistical analyses were performed using SPSS software (SPSS 22.0, SPSS, Inc., Chicago, IL). All parameters were expressed as mean ± standard deviation. The Kolmogorov-Smirnow test was used to determine normality of distribution. Mann-Whitney U test was used to compare the parametric data between groups and student t test was used to compare the non-parametric data between groups. A value of p < 0.05 was considered statistically significant.

## Results

**Figure 1:**
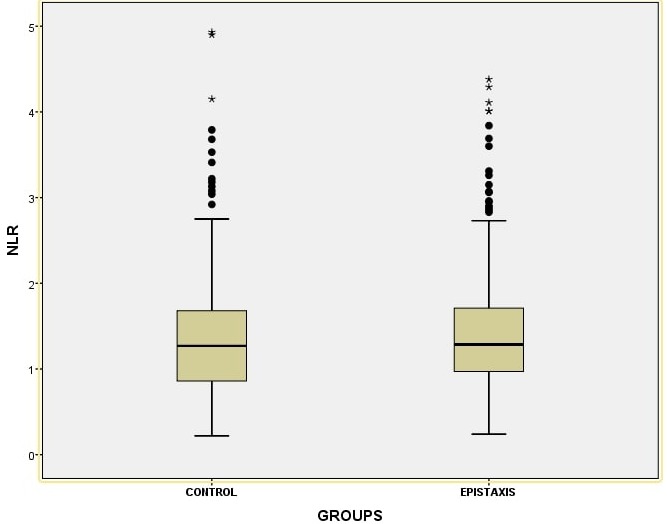
the mean Neutrophil to lymphocyte ratio (NLR) values of the epistaxis group and control group

**Figure 2:**
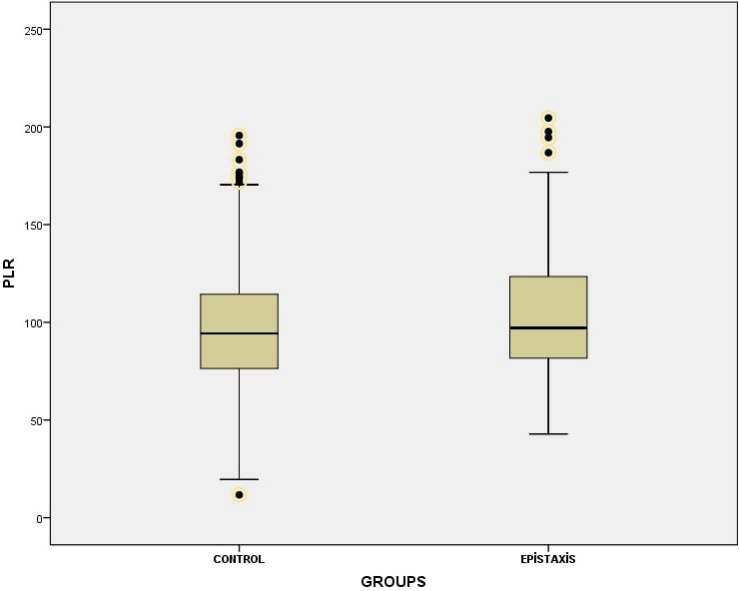
the mean Platelet to lymphocyte ratio (PLR) values of the epistaxis group and the control group

Of the 294 patients in the pediatric epistaxis group, 145 (49.31%) were male and 149 (50.69%) were female. Of the 329 children in the control group, 160 (48.63%) were male and 169 (51.37%) were female. There was no statistically significant difference between pediatric recurrent epistaxis and control group in terms of mean age and gender distribution. The demographic characteristics of the study and control groups are shown in Table 1. When the blood parameters were evaluated, there was no statistical difference between the two groups in terms of mean NLR values, whereas it was found that the mean PLR value was statistically higher in the pediatric recurrent epistaxis group compared to the control group (p=0.11, p=0.025, respectively) (Figure 1, Figure 2).

**Table 1:** the demographic and clinical features of the Study populations (Patients and Controls)

Variables	Control Group (n=329)	Study group (n=296)	p
	Mean±SD	Mean±SD	
Age	9±4.78	9,16±4.23	0.338
**Sex**			
Male	160	145	
Female	169	149	
NLR	1.35±0.7	1.45±0.75	0.11
PLR	97.3±30.38	103.21±29.57	0.025
Hemoglobin g/dL	13.19±1.45	12.92±1.27	0.004
HCT %	39.62±4.15	38.74±3.62	0.003
MCV fL	81.55±6.02	80.72±4.96	0.009
MCH pg	27.17±2.33	26.92±1.91	0.038
MCHC g/dL	35.88±26.88	35.4±24.79	0.569
RDW %	39.56±2.87	38.92±2.46	0.004
PLT 103 /u	278.43±57.99	293.15±56.12	0,001
MPV fL	9.45±1.02	9.41±0.93	0.566
PCT %	0.65±0.87	0.61±0.86	0.444
PDW %	15.36±1.37	15.08±1.81	0.104
WBC 10^3^ /u	7.59±1.57	7.81±1.87	0.115
Neutrophil %	48.63±10.92	50.41±10.99	0.043
Lymphocytes %	40.74±10.58	39.52±10.24	0.143
Monocytes %	6.53±1.66	6.49±1.82	0.499
Eosinophils %	3.6±2.95	3.23±2.68	0.099
Basophil, %	0.39±0.22	0.37±0.2	0.256
Neutrophil 10^3^ /u	3.73±1.29	4.03±1.57	0.029
Lymphocytes,	3.08±0.98	3.02±0.85	0.647
Monocytes 10^3^ /u	0.49±0.15	0.5±0.16	0.926
Eosinophils 10^3^ /u	0.28±0.24	0.26±0.23	0.148
Basophil 10^3^ /u	0.03±0.02	0.03±0.02	0.229

SD, Standart Deviation; HCT, hematocrit; MCH, mean corpuscular hemoglobin; MCHC, mean corpuscular hemoglobin concentration; MCV, mean corpuscular volume; MPV, mean platelet volume; NLR, neutrophil-to-lymphocyte ratio; PCT, platelet crit; PDW, platelet distribution width; PLR, platelet-to-lymphocyte ratio; PLT, platelet; RBC, red blood cell; RDW, red cell distribution width; WBC, white blood cell. p< 0.05 value was regarded as significant. The significant differences between the groups are shown in bold.

* Student t test

** Mann–Whitney U test

In addition, it was found that the mean neutrophil count (p=0.029), percentage of neutrophils (%) (p=0.043), platelet count (PLT) (p=0.001) and PLR value (p=0.025) were statistically higher in the recurrent epistaxis group compared to the control group (Table 1). It was found that Hemoglobin (Hgb) (p=0.004), Hematocrit (Hct) (p=0.003), Mean Corpusculer hemoglobin (MCH) (p=0.038), Mean Corpusculer Volume (MCV) (p=0.009) and Red cell distribution width (Rdw) (p=0.004) was statistically significantly lower in the recurrent epistaxis group than the control group. On the other hand, it was found that there was no statistical significant difference between the two groups in terms of Basophil count, percentage of Basophil (%), Eosinophil count, percentage of Eosinophil (%), Lymphocyte count, percentage of Lymphocyte (%), Mean Corpusculer Hemoglobin Concentration (MCHC), Monocyte count, percentage of Monocyte (%), Mean Platelet Volume (MPV), Platelet crit (PCT), Platelet distribution width (PDW) values (p>0.05) (Table 1).

## Discussion

One of the most important results of this study is that the PLR value was found to be significantly higher in the pediatric recurrent epistaxis group compared to the control group. However, there was no statistical difference between the groups in terms of NLR value that is another inflammatory marker. It was found that Neutrophil count, percentage of neutrophil, Platelet count were statistically significantly higher in the study group than the control group. To our knowledge, this present study was the first study to investigate NLR and PLR in pediatric recurrent epistaxis. One of the other important results of our study is that the Hgb, HCT, MCH, MCV and RDW levels were found to be significantly lower in the study group than the control group.

Epistaxis is one of the most common medical conditions in childhood and pediatric epistaxis is one of the most common causes of otolaryngology admissions [1]. Digital trauma, medication, dehumidification, septal perforation, neoplasm, hereditary hemorrhagic telangiectasia, congenital bleeding disorders are among the causes of pediatric epistaxis [13]. In addition, nasal bacterial colonization (especially *Staphylococcus aureus*) factor was asserted in the etiology of pediatric recurrent epistaxis [10]. The reduction in bleeding frequency by antiseptic creams in children with recurrent epistaxis, supports nasal bacterial colonization theory [14]. Complete blood count is an inexpensive method used routinely. It has an important place in the clinic because it gives information in terms of the white and red blood cell count, platelet count, MPV, RDW levels and the parameters such as NLR and PLR [5].

The most important indicator of inflammation in CBC is the increase in the count of leukocytes and its subtypes. Neutrophils are the first defense cells of the immune system and contribute to phagocytosis and apoptosis through mediators secreted by them. Lymphocytes constitute the protective and regulating part of immunity [15]. NLR comes to the forefront as a biomarker used to investigate the role of inflammation in different diseases [5, 16]. It was found that the elevated NLR and PLR values were associated with poorer outcome and increased inflammatory process in some autoimmune diseases, acute pancreatitis, and in some malignant diseases [17]. In another study, it was found that the NLR values in infective endocarditis were higher in patients with high hospital mortality compared to patients with low hospital mortality [18]. In our study, while neutrophil count was found to be significantly higher in the study group than in the control group, no difference was found between the two groups in terms of NLR. Increased neutrophil count supports the possible role of inflammation in pediatric recurrent epistaxis.

PLR was used as an indicator of systemic inflammatory response in recent years, although not as frequent as NLR. The expression of inflammatory cytokines increase with the increased platelet count under the inflammatory condition and in case of bleeding [5]. In previous studies, the increased PLR values were found in systemic lupus erythematosus, psoriasis vulgaris and psoriatic arthritis [19, 20]. There is no study performed to investigate PLR levels in epistaxis. On the other hand, there are three studies about the change of MPV, which is an indicator of the size of the platelets and thus hemostatic activity in epistaxis. In the study of Kemal *et al.* it was detected that MPV values were statistically lower in the epistaxis group compared to the control group [21]. Similarly, in the study of Karabulut *et al.* it was found that MPV values were statistically significantly lower in the study group than in the control group [22]. However, Bezgin *et al.* found no statistically significant difference between the study group and the control group in terms of MPV value in their study with pediatric epistaxis patients [11]. In our study, MPV was found to be 9.41±0.93 in the study group and 9.45±1.02 in the control group. However, there was no statistically significant difference in terms of MPV. In the present study, the relationship between high PLR value and epistaxis brings to mind the inflammatory factors in the pathogenesis of epistaxis. The results of the present study and with three previous studies show that there is no complete consensus about the change of MPV in epistaxis. In future, the studies on the change of MPV in epistaxis may reveal the usability of MPV in prognosis.

RDW is a test that is easily accessible, inexpensive and found in routine hemogram assay. It was often used to investigate the cause of anemia. In previous studies, RDW was investigated in terms of the risk and prognosis of the diseases such as heart failure, hepatocellular carcinoma [23, 24]. In the present study, we found that RDW levels were significantly lower in study group than the control group. This result was correlated with the study in adults by Kemal *et al.* and with the study in children by Bezgin *et al.* [11, 21]. On the other hand, in the study by Karabulut *et al.* of adult patients with epistaxis, RDW values were found to be significantly higher in study group than the control group. Bezgin *et al.* asserted that decreased RDW level in recurrent epistaxis was due to its anti-thrombotic activity and the recurrent epistaxis may occur because of this reason [11]. Salvagno *et al.* asserted that the increase of RDW levels due to increased bleeding stress were an expected result [25]. In contrast, the decreased RDW values in epistaxis found in our study may provide RDW to be used as a marker.

## Conclusion

In our study, there was no correlation between NLR and recurrent epistaxis, whereas PLR values were found to be higher in epistaxis group. Low RDW values were found in the recurrent epistaxis group. According to the results of present study, the increase of PLR, an inflammatory marker, in epistaxis supports the effect of inflammatory factors in the etiology of epistaxis. However, more study in future is needed to support this.

### What is known about this topic

- Inflammation can cause epistaxis in children;
- Epistaxis is usually seen in pediatric patients;
- Epistaxis is usually idiopathic.

### What this study adds

- Neutrophil lymphocyte ratio (NLR) and platelet to lymphocyte ratio (PLR) was studied first in this study according to previous literature;
- Results of this study is showing that inflammation can play a role in pediatric epistaxis;
- This study include very wide patient group according previous literature.

## Competing interests

The authors declare no competing interests.
